# Construction of a UBE2S‐ and Ferroptosis‐Based Prognostic Model for Hepatocellular Carcinoma and Validation of UBE2S‐Mediated Ferroptosis

**DOI:** 10.1155/humu/2592640

**Published:** 2026-11-08

**Authors:** Hao Zhang, Jiajun Yao, Jun Cao, Zehao Guo, Wendong Jiang, Kangzhi Li, Xiaoyan Wang, Zhijing Mo

**Affiliations:** ^1^ Key Laboratory of Cell and Gene Therapy for Regional High-incidence Diseases Education Department of Guangxi Zhuang Autonomous Region Guilin Medical University Guilin China glmc.edu.cn; ^2^ Department of Biochemistry College of Basic Medical Sciences Guilin Medical University Guilin China glmc.edu.cn

**Keywords:** ferroptosis, hepatocellular carcinoma, prognosis, sorafenib

## Abstract

**Purpose:**

This study is aimed at exploring whether ubiquitin‐conjugating enzyme E2 S (UBE2S) influences hepatocellular carcinoma (HCC) progression through ferroptosis and at constructing a UBE2S‐related ferroptosis prognostic model (UFP) for predicting clinical outcomes in HCC patients.

**Methods:**

Bioinformatics approaches were employed to examine how UBE2S expression relates to both HCC prognosis and ferroptosis. Tumor immune infiltration was assessed using CIBERSORT with the LM22 signature matrix. The prognostic accuracy of the UFP, which integrates CENPA and PAGE1, was assessed via survival analysis, ROC curves, and calibration plots. For experimental validation, ferroptosis‐related markers were measured following UBE2S overexpression or knockdown, or after treatment with the ferroptosis inducers erastin and sorafenib. Additionally, tumor weight, size, and ferroptosis‐associated proteins were examined in a xenograft model following combined UBE2S knockdown and sorafenib treatment.

**Results:**

In HCC, elevated UBE2S expression predicted poor prognosis, suppressed ferroptosis, and correlated with an immunosuppressive microenvironment. The UFP model demonstrated robust prognostic performance, as validated using ICGC and single‐cell datasets. UBE2S knockdown promoted ferroptosis, as shown by increased Fe^2+^, MDA, and ROS levels and decreased GSH, whereas UBE2S overexpression reversed erastin‐induced ferroptosis. Moreover, UBE2S knockdown downregulated GPX4 and SLC7A11, whereas UBE2S overexpression upregulated these proteins. Sorafenib reduced UBE2S levels, and UBE2S overexpression reversed sorafenib‐induced ferroptosis. In xenograft models, UBE2S knockdown combined with sorafenib reduced tumor growth and enhanced ferroptosis.

**Conclusion:**

The UFP model serves as a novel prognostic biomarker for HCC outcomes. Mechanistically, UBE2S suppression promotes ferroptosis and inhibits tumor progression, highlighting this protein as a promising therapeutic candidate. Collectively, our results offer fresh perspectives on HCC management strategies.

## 1. Introduction

Globally, hepatocellular carcinoma (HCC) ranks as one of the deadliest malignancies, accounting for 70%–80% of liver cancer cases worldwide [1, 2]. Even with advances in detection and therapy, the prognosis of HCC remains poor, with only 10%–18% survival at 5 years [3]. To advance patient survival and quality of life in HCC, novel biomarkers must be discovered and validated for diagnosis and prognosis, alongside continued development of targeted therapies [4, 5].

Ferroptosis is a distinct type of regulated cell death marked by the build‐up of lipid peroxides, whose molecular mechanisms are fundamentally connected to cellular redox homeostasis and metabolic pathways involving cysteine [6, 7]. As a selenoprotein within the glutathione peroxidase (GPX) family, glutathione peroxidase 4 (GPX4) exerts crucial biological functions in cellular antioxidant defense systems. The conversion of cytotoxic lipid hydroperoxides into less harmful products by this enzyme relies on reduced glutathione (GSH) as an essential cofactor, which also contributes to redox homeostasis and cysteine metabolic regulation [8]. Loss of GPX4 function triggers ferroptosis through the accumulation of lipid peroxides [9]. Recent studies have elucidated distinct molecular pathways modulating ferroptosis in cancer progression. Li et al. demonstrated that CST1 modulates GPX4 ubiquitination status through interaction with OTUB1, establishing a protective mechanism against ferroptotic cell death that facilitates gastric cancer metastasis [10]. Complementing this finding, Hu et al. identified ASS1‐mediated glutamine reductive carboxylation as a metabolic switch that suppresses lipid peroxidation, effectively protecting cancer cells from ferroptosis [11]. These mechanistic insights have propelled ferroptosis regulation to the forefront of oncological research, with targeted modulation of ferroptotic pathways now being actively explored as a promising therapeutic paradigm [12, 13]. Although there have been advances in diagnostics and therapies, the outcome for patients with advanced‐stage HCC stays poor, because only about a third can receive effective treatments [14]. Key markers include Golgi protein‐73 (GP73) for differential diagnosis [15], squamous cell carcinoma antigen (SCCA) isoforms monitoring tumor progression [16], and des‐*γ*‐carboxy prothrombin (DCP) detecting early lesions and predicting vascular invasion [17]. These biomarkers collectively enhance HCC management through complementary diagnostic and prognostic insights. However, models combining multiple biomarkers are not yet perfect and require further research [18]. Clinically, late‐stage HCC therapeutic drugs such as sorafenib, lenvatinib, and artemisinin can promote ferroptosis in HCC cells, thus exerting anticancer effects. Therefore, identifying new ferroptosis‐related biomarkers to construct prognostic models will aid in the assessment of HCC patient prognosis.

The ubiquitin‐proteasome cascade mediates controlled protein degradation through three enzymatic steps: E1 activating enzymes initiate ubiquitin activation via ATP hydrolysis, E2 conjugating enzymes transfer the activated ubiquitin, and E3 ligases specifically recognize substrates while catalyzing ubiquitin ligation. This hierarchical enzymatic architecture ensures precise regulation of proteolytic targeting. The interplay between these enzymes is crucial for oncogenesis, influencing the stability and degradation of proteins that can lead to cancer [19, 20]. The functional repertoire of ubiquitin enzymes is essential in regulating various aspects of protein behavior, including their subcellular localization, susceptibility to degradation by the proteasome, and participation in signaling cascades, which in turn significantly shape cellular processes and outcomes [21]. Ubiquitin‐conjugating enzyme E2 S (UBE2S) belongs to the family of enzymes responsible for ubiquitin conjugation, known as E2 ubiquitin‐conjugating enzymes. An increasing amount of evidence indicates that UBE2S influences the progression of many types of cancers [22, 23]. Mechanistically, UBE2S promotes HCC development by driving cell cycle progression via p27 ubiquitination [24] and confers resistance to 5‐FU and oxaliplatin through PTEN‐AKT pathway activation [25]. In addition, this ubiquitin‐conjugating enzyme enhances HCC cell proliferation and migration while promoting p53 ubiquitination, collectively underscoring its oncogenic role [26]. Notably, p53 itself mediates ferroptosis to suppress tumor growth [27]. Therefore, based on the above research findings, UBE2S may be related to ferroptosis in HCC cells. However, there is currently no research elucidating the impact of UBE2S on ferroptosis in HCC, warranting further investigation.

Therefore, the relationship of UBE2S with HCC prognosis and UBE2S with ferroptosis was analyzed in this study. Through integrated bioinformatics approaches, we identified optimal ferroptosis‐related prognostic biomarkers for developing the UFP prognostic signature. Multivariate validation confirmed the UFP score′s independence from conventional prognostic factors, with subsequent development of a clinical prediction nomogram. The ICGC and single‐cell datasets were used as external validation sets. Furthermore, we elucidated the regulatory effect of ferroptosis inducer erastin and sorafenib on UBE2S expression patterns and established through in nude mouse models the functional impact of UBE2S modulation on tumor progression metrics and ferroptotic pathway activation. This research seeks to identify a reliable biomarker for predicting HCC prognosis and aims to establish a theoretical foundation for advancing UBE2S research.

## 2. Materials and Methods

### 2.1. HCC Sample Data Source and Format

We extracted transcriptomic (HTSeq‐Counts) and clinicopathological data for 357 HCC and 44 normal liver tissues from the TCGA‐LIHC database, including pathological stage, T stage, G stage, age, sex, and survival outcomes. Samples that lacked clinical data were excluded. HTSeq‐Counts were transformed using Log2 (TPM+1) for subsequent analyses, and the same count data were used for differential expression analysis.

### 2.2. UBE2S Prognostic Analysis

UBE2S expression levels were compared between HCC and normal tissues. Patient cohorts were stratified based on UBE2S expression, and their survival outcomes were compared. Calibration curves and the area under the curve (AUC) were used to assess the prognostic performance of UBE2S.

### 2.3. Immune Infiltration Analysis by CIBERSORT

The fractions of 22 immune cell populations were quantified via the CIBERSORT algorithm (R package v1.03) based on the LM22 signature matrix with 1000 permutations. The resulting immune cell fraction matrix was used to compare infiltration patterns across the two expression groups.

### 2.4. Ferroptosis Feature Evaluation

Key regulators of ferroptosis were identified via the FerrDb database, and *t*‐tests were performed to assess differential expression of these genes between groups. The single‐sample gene set enrichment analysis (ssGSEA) algorithm from the GSVA R package was applied to quantify ferroptosis‐related pathway enrichment in HCC samples, using gene sets obtained from MSigDB and WikiPathways.

### 2.5. Detection of Differentially Expressed Genes (DEGs)

To identify DEGs, we applied DESeq2 with a cutoff of |log2FC| > 1 for expression change and adjusted *p* < 0.05 for significance.

### 2.6. Construction of UFP

The survival and glmnet R packages facilitated LASSO regression on 675 candidate genes to identify prognostic markers. A prognostic model, designated UFP, was constructed with the risk score defined as $iskScore=\sum_{k=0}^{n}\beta i\times Expi$ . Using the median as the cutoff, patients were split into high‐ and low‐risk groups for subsequent survival analysis.

### 2.7. Development and Validation of HCC Prognostic Nomogram

To enhance clinical applicability, we integrated the UFP risk score with significant clinicopathological variables to construct a prognostic nomogram. Predictive performance was assessed via the rms R package.

### 2.8. Validation Using External Datasets From ICGC

To establish an external validation set, we obtained RNA‐sequencing profiles (HTSeq‐Counts) and clinical annotations from 497 HCC patients in the ICGC repository.

### 2.9. Single‐Cell RNA Sequencing Analysis of UFP

Quality control, normalization, and clustering of single‐cell RNA‐seq data from HCC and normal liver specimens (GSE149614) were carried out with the Seurat and Harmony packages. Using the clusterProfiler R package, we conducted GO and KEGG analyses on the identified cell clusters.

### 2.10. Cell Culture and Transfection

Huh7 and Hep3B cells were cultured in DMEM containing 10% FBS and 1% penicillin–streptomycin. Lentiviral particles for UBE2S knockdown were transduced into the cells, followed by puromycin selection. Table 1 presents the shRNA oligonucleotide sequences targeting UBE2S.

**Table 1:** UBE2S knockdown sequences.

ShRNA construct	Sequence
LV1‐UBE2S knockdown sequence	5 ^′^‐GCTACTTCCTGACCAAGATCT‐3 ^′^
LV2‐UBE2S knockdown sequence	5 ^′^‐GACACGTACTGCTGACCATCA‐3 ^′^
LV3‐UBE2S knockdown sequence	5 ^′^‐GGAGGTCTGTTCCGCATGAAA‐3 ^′^

### 2.11. Ferroptosis Assays

For UBE2S overexpression, cells received either a UBE2S‐expressing construct (OE‐UBE2S) or an empty vector (Vector). Ferroptosis was induced by exposing cells to 10–20‐*μ*M erastin or 10‐*μ*M sorafenib for 24–48 h as specified. Following these treatments, commercially available kits (Abbkine) were used to quantify intracellular levels of ferrous iron (Fe^2+^), malondialdehyde (MDA), GSH, and reactive oxygen species (ROS) according to the manufacturer′s protocols. All assays were run in triplicate.

### 2.12. Western Blot Analysis

Protein lysates prepared in RIPA buffer with inhibitors (Thermo Fisher) were subjected to SDS‐PAGE, transferred to PVDF membranes (Millipore), and blocked. Blots were probed with anti‐UBE2S (1:500), anti‐SLC7A11 (1:1000), anti‐GPX4 (1:1000), and anti‐*β*‐actin (1:3000), followed by HRP‐secondary antibody (1:10,000) (all from Abcam). Signals were detected by ECL.

### 2.13. Xenograft Model

Nude mice (6–8 weeks old, male) were injected subcutaneously with 1 × 10^6^ Hep3B cells. Once neoplasms reached approximately 100 mm^3^, the animals were randomly assigned to four treatment cohorts: control (LV‐shNC), sorafenib (20 mg/kg twice daily), LV‐shUBE2S1, and LV‐shUBE2S1 combined with sorafenib. Tumor dimensions and weights were recorded, and tissues were subsequently harvested for immunohistochemistry (IHC).

### 2.14. IHC

Tissue sections (4 *μ*m) underwent deparaffinization, rehydration, and antigen retrieval (citrate buffer, pH 6.0, 95°C), with endogenous peroxidase activity subsequently quenched using 3% H₂O₂. Sections were incubated with primary antibodies against UBE2S (1:100), SLC7A11 (1:500), GPX4 (1:100), ACSL4 (1:100), and TFRC (1:100) (all from Abcam), followed by HRP‐conjugated secondary antibody (1:1000) and DAB development.

### 2.15. Statistical Analysis

Each experiment was carried out three times independently. GraphPad Prism 8.0 was used for data analysis, with ANOVA and *t*‐tests applied for group comparisons. Statistical significance was defined as *p* < 0.05.

## 3. Results

### 3.1. UBE2S Upregulation Predicts Adverse HCC Outcomes

Compared with normal liver tissues, HCC specimens exhibited significantly elevated UBE2S expression (*p* < 0.001) (Figure 1A). Notably, variables including patient gender and age showed no significant correlation with UBE2S expression patterns (Figure 1B–C). In terms of tumor staging (T stage), the expression of UBE2S was markedly increased in T3_4 tumors relative to those in T1_2 stages (Figure 1D). Similarly, when examining pathological staging, UBE2S expression was observed to be considerably higher in advanced stages III_IV as opposed to the earlier stages I_II (Figure 1E). Regarding G stage, UBE2S expression was significantly higher in stages G3_4 compared to G1_2 (Figure 1F). Patients with HCC in High‐UBE2S group exhibited worse prognosis (*p* < 0.05) (Figure 1G). The predictive performance of UBE2S for 1‐, 2‐, and 3‐year survival was reflected in AUCs of 0.72, 0.70, and 0.67, as determined by ROC analysis (Figure 1H). Moreover, calibration curves showed strong concordance between predictions and actual observations, confirming UBE2S as a robust prognostic marker (Figure 1I).

Figure 1UBE2S upregulation predicts adverse HCC outcomes. (A) UBE2S expression in HCC versus normal controls. (B) Gender‐specific UBE2S expression patterns. (C) Age‐related UBE2S expression levels. (D) UBE2S expression by tumor stage (T stage). (E) UBE2S expression by pathological stage. (F) UBE2S expression by histological grade (G stage). (G) Survival outcomes by UBE2S expression. (H) Time‐dependent ROC analysis. (I) Calibration of predicted survival probabilities. (J) Immune cell infiltration fractions across UBE2S expression groups. (K) UBE2S expression in cell clusters identified from single‐cell analysis. Asterisks indicate *p*‐value thresholds: ns, not significant; ∗*p* < 0.05; ∗∗*p* < 0.01; ∗∗∗*p* < 0.001.

**Figure:**
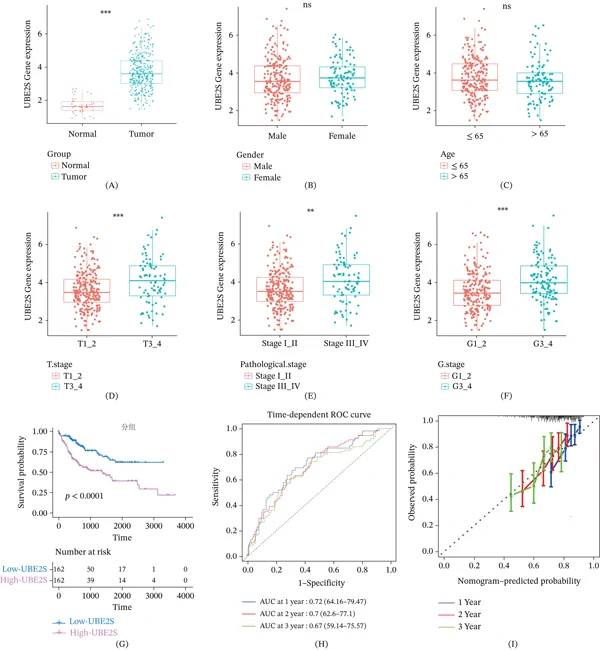

**Figure:**
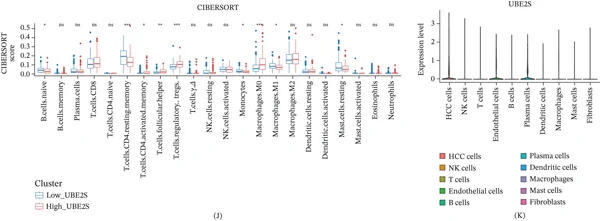

Furthermore, our bioinformatic analysis using the CIBERSORT algorithm revealed predictive correlations between UBE2S expression and the abundance of 22 immune cell types in HCC (Figure 1J). Significant differences were observed in several cell populations. The high‐UBE2S group exhibited a reduced fraction of resting memory CD4^+^ T cells and an elevated fraction of Tregs and M0 macrophages (*p* < 0.001). Follicular helper T cells showed a highly significant difference (*p* < 0.01), whereas naive B cells, M1 macrophages, resting mast cells, monocytes, and activated memory CD4^+^ T cells displayed significant differences (*p* < 0.05). The remaining cell types showed no significant differences. In single‐cell data, UBE2S was enriched in HCC cells, endothelial cells, and plasma cells (Figure 1K). These findings represent computational predictions of correlative patterns between UBE2S expression and immune cell infiltration, rather than functionally validated relationships.

### 3.2. UBE2S Is Associated With Ferroptosis Suppression in HCC

High‐ and low‐UBE2S groups were compared for the expression of redox‐related, lipid‐related, GSH‐related, iron‐related, and other regulatory gene sets. Within the redox‐related gene set, antiferroptotic genes including GPX4, AIFM2, HMOX1, CISD1, and HIF1A were notably overexpressed in the High‐UBE2S group, whereas pro‐ferroptotic genes including PEBP1 and DPP4 showed marked upregulation in the Low‐UBE2S group (Figure 2A). In the lipid‐related gene set, antiferroptotic genes (HELLS and FADS2) were markedly elevated in the High‐UBE2S group (Figure 2B). In the GSH‐related gene set, antiferroptotic genes (SLC7A11 and MUC1) were markedly elevated in the High‐UBE2S group, whereas the pro‐ferroptotic gene GLS2 was markedly upregulated in the Low‐UBE2S group (Figure 2C). In the iron‐related gene set, the High‐UBE2S group exhibited significant upregulation of antiferroptotic genes (FTL, FTH1, and PCBP2), whereas pro‐ferroptotic genes (TFRC, SLC39A14, and STEAP3) were markedly elevated in the Low‐UBE2S group (Figure 2D). Regarding the other regulatory gene sets, antiferroptotic genes (PHGDH, G6PD, ME1, ATF4, TAZ, and FANCD2) were markedly elevated in the High‐UBE2S group, whereas the proferroptotic gene ATF3 was markedly upregulated in the Low‐UBE2S group (Figure 2E). The High‐UBE2S group showed lower ssGSEA scores for ferroptosis and oxidative stress (Figure 2F–G), suggesting a suppressive role of UBE2S in ferroptosis.

**Figure 2:**
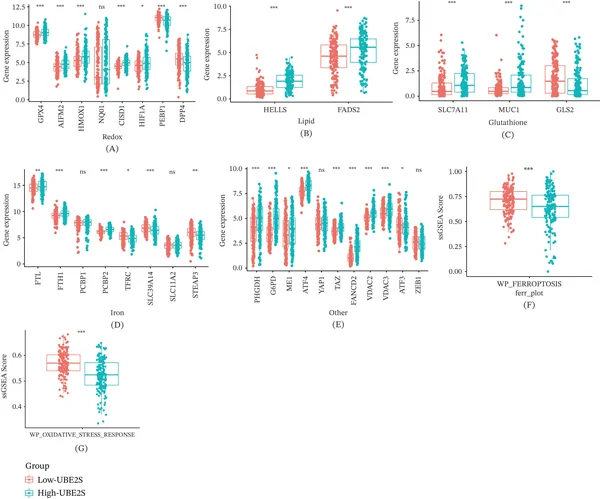
UBE2S is associated with ferroptosis suppression in HCC. (A–E) Expression of redox, lipid, glutathione, iron, and other regulatory genes in High‐UBE2S versus Low‐UBE2S groups. (F) ssGSEA scores for ferroptosis‐related features in the two groups. (G) ssGSEA scores for oxidative stress‐related features in the two groups. Asterisks indicate *p*‐value thresholds: ns, not significant; ∗*p* < 0.05; ∗∗*p* < 0.01; ∗∗∗*p* < 0.001.

### 3.3. Construction of the UBE2S‐Related Ferroptosis Prognostic Model (UFP)

By intersecting ferroptosis‐related genes (FERR_genes), HCC‐related genes (HCC_genes), and UBE2S‐related genes (UBE2S_genes), 675 DEGs (UFP_DEGs) were obtained (Figure 3A). LASSO regression, together with univariate and multivariate Cox analyses, was applied to these 675 genes to screen for prognostic genes (Figure 3B–D). Ultimately, two independent prognostic genes, namely CENPA and PAGE1, were selected to construct the UFP. The UFP risk score was formulated as 0.3640172 × CENPA +0.1202112 × PAGE1. UBE2S, CENPA, and PAGE1 transcript levels were positively correlated (Figure 3E). High‐UFP patients exhibited markedly worse survival outcomes (Figure 3F). ROC analysis for 1‐, 2‐, and 3‐year survival yielded AUCs of 0.75, 0.71, and 0.70, confirming the predictive accuracy of the UFP (Figure 3G). Calibration curves showed that the predicted values closely approached the actual values (Figure 3H), indicating that the UFP exhibited robust performance for survival prediction in HCC.

**Figure 3:**
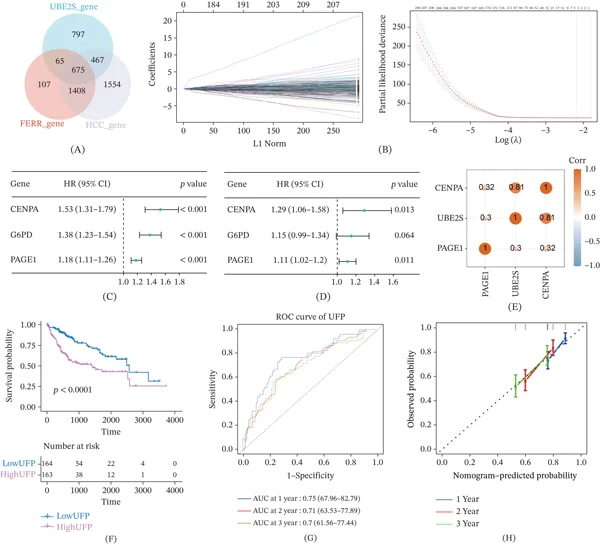
Construction of the UFP. (A) UFP_DEGs obtained from the intersection of UBE2S_genes, FERR_genes, and HCC_genes. (B) LASSO regression for initial screening of UBE2S‐ferroptosis–related prognostic genes. (C) Univariate Cox proportional for preliminary identification of prognostic factor. (D) Multivariate Cox regression for establishing independent prognostic variables. (E) Pairwise correlation matrix of UFP components, with color gradients (red: positive; blue: negative) reflecting Pearson′s correlation coefficient. (F) Kaplan–Meier survival curves for LowUFP and HighUFP groups. (G) ROC curves for UFP prognostic performance. (H) Calibration of predicted survival probabilities.

### 3.4. UFP and Pathological Stage for Prognostic Prediction: Development of a Nomogram

In prognostic factor analysis using Cox regression models (both univariate and multivariate approaches), pathological staging and UFP emerged as independent predictors of HCC outcomes (Figure 4A–B). The predictive validity assessment through ROC curves yielded 3‐year AUC metrics of 0.48 (age), 0.55 (gender), 0.53 (G stage), and 0.64 (pathological stage), with the highest predictive accuracy observed for pathological staging (Figure 4C). Notably, the UFP demonstrated a 3‐year AUC value of 0.70, exceeding those of the aforementioned clinical features. For 1‐, 2‐, and 3‐year survival, the combination of UFP and pathological stage produced the highest predictive performance, with AUCs of 0.78, 0.73, and 0.76 (Figure 4D). Good concordance between predictions and actual outcomes was revealed by the calibration curves (Figure 4E). Based on UFP and pathological staging parameters, we constructed a prognostic nomogram for HCC outcome prediction (Figure 4F).

**Figure 4:**
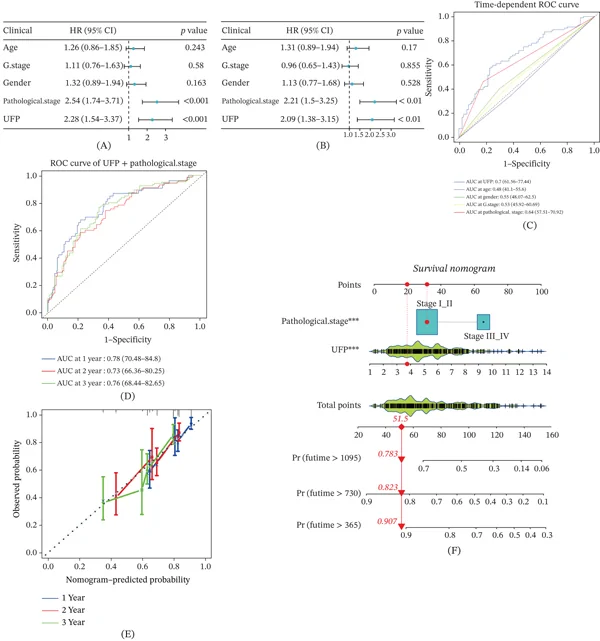
Nomogram integrating UFP and pathological stage for prognosis prediction in HCC. (A) Univariate Cox regression for survival‐associated factors. (B) Evaluation of independent prognostic markers via multivariate Cox regression. (C) ROC‐based AUC analysis of clinical factors. (D) ROC‐based AUC analysis combining UFP and pathological stage. (E) Calibration of predicted survival probabilities. (F) Nomogram integrating UFP and pathological stage. Asterisks indicate *p*‐value thresholds: ∗∗∗*p* < 0.001.

### 3.5. Validation Using External Datasets From the ICGC

We employed ICGC data to validate the TCGA dataset′s outcomes, confirming no significant UBE2S expression variances by age or gender (Figure 5A–B). Notably, UBE2S expression was markedly elevated in pathological stages III_IV versus I_II (Figure 5C). The 497 HCC ICGC samples, stratified by UFP score into LowUFP and HighUFP groups, showed that the HighUFP group had a significant correlation with poorer prognosis (*p* < 0.001) (Figure 5D). The UFP achieved 1‐, 2‐, and 3‐year AUCs of 0.76, 0.79, and 0.74, outperforming age (0.48), gender (0.51), and pathological stage (0.67) at 3 years (Figure 5E–F). Integrating the UFP with pathological stage yielded superior predictive performance, with 1‐, 2‐, and 3‐year AUCs of 0.87, 0.78, and 0.75, highlighting its accuracy and robustness in predicting outcomes (Figure 5G). Calibration curves confirmed close agreement between predicted and observed survival probabilities (Figure 5H), consistent with TCGA dataset analyses.

**Figure 5:**
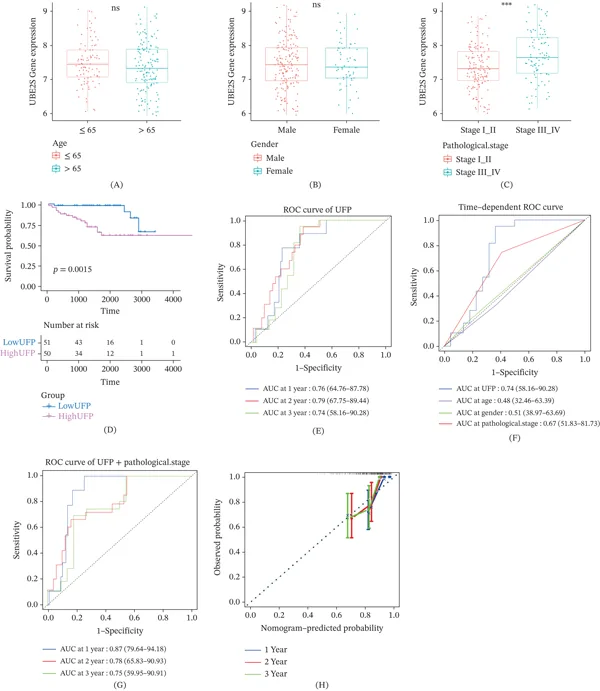
Validation using external datasets from ICGC. (A) UBE2S expression by age group. (B) UBE2S expression by gender. (C) UBE2S expression by pathological stage. (D) Kaplan–Meier survival curve for LowUFP and HighUFP groups. (E) ROC‐based AUC analysis of clinical factors. (F) ROC‐based AUC analysis of clinical factors. (G) ROC‐based AUC analysis combining UFP and pathological stage. (H) Calibration of predicted survival probabilities. Asterisks indicate *p*‐value thresholds: ns, not significant; ∗∗∗*p* < 0.001.

### 3.6. Single‐Cell Data Analysis of UFP

To assess UFP expression patterns at single‐cell resolution, we interrogated HCC‐derived scRNA‐seq datasets. Clustering of cells from 10 HCC and 8 normal samples yielded 45 cell clusters (Figure 6A). Through marker‐based stratification, these clusters were systematically classified into 10 major lineages: macrophages, malignant/normal hepatocytes, NK cells, CD3+ T lymphocytes, dendritic cells (DCs), vascular endothelial cells, B cells, fibroblasts, mast cells, and plasma cells (Figure 6B–C). Two subsets were classified based on the UFP risk score: a high‐risk subset (HCC_UFP) and a low‐risk subset (HCC) (Figure 6D–E). The proportion of the HCC_UFP subset was significantly higher in stages 3_4 HCC cells compared to stages 1_2 HCC cells (Figure 6F). ssGSEA scores for ferroptosis and oxidative stress were substantially greater in the HCC subset compared to the HCC_UFP subset (*p* < 0.001) (Figure 6G–H). GO enrichment analysis revealed significant enrichment of ubiquitin protein ligase binding and GTPase activity within the HCC_UFP subpopulation, whereas the HCC subset was mainly associated with lipid transporter activity and steroid dehydrogenase activity (Figure 6I). KEGG pathway analysis showed that the HCC_UFP subset was predominantly enriched in proteasome and cell cycle pathways, while the HCC subset was mainly associated with peroxisomes, mTOR signaling pathway, and ferroptosis pathways (Figure 6J). Differential expression analysis of ferroptosis markers revealed that the HCC_UFP subset exhibited significantly higher expression of the antiferroptotic genes GPX4, CISD1, PCBP1, PCBP2, and ATF4, whereas the proferroptotic genes PEBP1 were significantly higher in the HCC subset (Figure 6K).

Figure 6Single‐cell data analysis of UFP. (A) UMAP clustering of 45 cell clusters. (B) Expression of characteristic markers across cell cluster. (C) UMAP clustering plot of 10 cell clusters. (D) Distribution of UFP risk score across 10 cell clusters. (E) Subset clustering plot of HCC cells. (F) Proportions of HCC_UFP and HCC subsets by clinical stage. (G) ssGSEA scores for ferroptosis‐related pathways between the two subsets. (H) ssGSEA scores for oxidative stress‐related pathways between the two subsets. (I) GO enrichment analysis comparing the two subsets. (J) KEGG pathway enrichment analysis comparing the two subsets. (K) Differential expression of ferroptosis marker genes between the two subsets. Asterisks indicate *p*‐value thresholds: ns, not significant; ∗∗∗*p* < 0.001.

**Figure:**
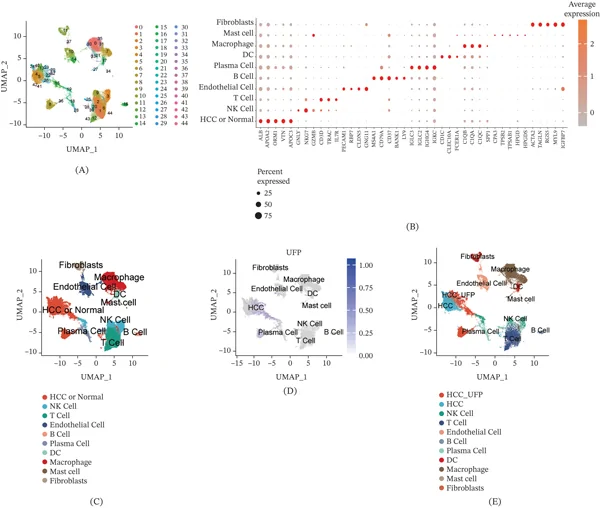

**Figure:**
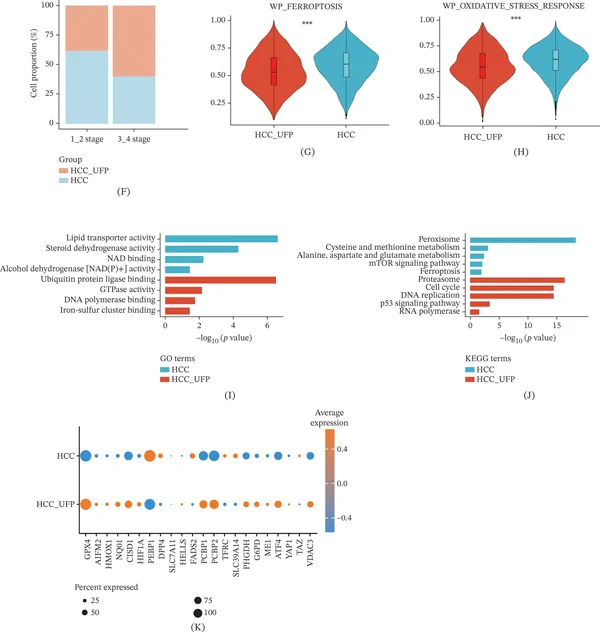

### 3.7. GPX4 and SLC7A11 Mediate UBE2S‐Induced Ferroptosis Suppression in HCC

UBE2S knockdown significantly elevated intracellular Fe^2+^ and MDA levels in Huh7 and Hep3B cells relative to the sh‐NC group (*p* < 0.001) (Figure 7A–B). Additionally, a marked elevation in ROS levels was observed in the sh‐UBE2S groups (*p* < 0.001) (Figure 7D). Conversely, GSH content was significantly decreased upon UBE2S silencing (*p* < 0.001) (Figure 7C). To further validate whether UBE2S suppresses ferroptosis, we performed rescue experiments using the ferroptosis inducer erastin in UBE2S‐overexpressing HCC cells. Erastin treatment markedly increased Fe^2+^, MDA, and ROS levels while decreasing GSH content in control cells (Vector + Erastin). However, overexpression of UBE2S significantly reversed the erastin‐induced alterations in these ferroptotic indicators (*p* < 0.001) (Figure 7E–H).

**Figure 7:**
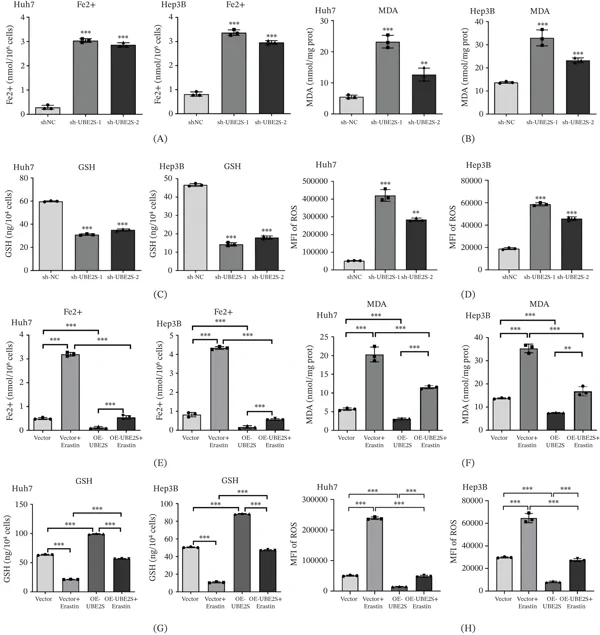
UBE2S inhibits ferroptosis in HCC cells. (A–D) Fe^2+^, MDA, GSH, and ROS levels in Huh7 and Hep3B cells with UBE2S knockdown (sh‐UBE2S‐1 and sh‐UBE2S‐2) versus control (sh‐NC). (E–H) Fe^2+^, MDA, GSH, and ROS levels in Huh7 and Hep3B cells treated with vector or OE‐UBE2S, with or without erastin. Asterisks indicate *p*‐value thresholds: ∗∗*p* < 0.01; ∗∗∗*p* < 0.001.

To investigate how UBE2S regulates ferroptosis, we examined GPX4 and SLC7A11, two canonical ferroptosis regulators. UBE2S knockdown markedly reduced GPX4 and SLC7A11 protein expression in both Huh7 and Hep3B cells relative to the sh‐NC group (Figure 8A,D). In contrast, UBE2S overexpression led to a pronounced increase in the protein levels of these two molecules (Figure 8B,E). qPCR analysis confirmed successful UBE2S overexpression at the mRNA level in the OE‐UBE2S group (*p* < 0.001) (Figure 8C,F).

**Figure 8:**
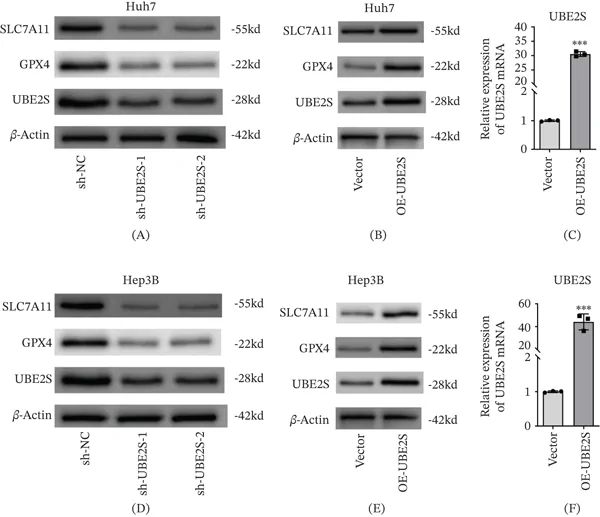
UBE2S regulates GPX4 and SLC7A11 expression in HCC cells. (A, D) UBE2S, GPX4, and SLC7A11 protein expression in (A) Huh7 and (D) Hep3B cells with UBE2S knockdown (sh‐NC, sh‐UBE2S‐1, and sh‐UBE2S‐2). (B, E) UBE2S, GPX4, and SLC7A11 protein expression in (B) Huh7 and (E) Hep3B cells with UBE2S overexpression (OE‐UBE2S) versus vector control. (C, F) Relative UBE2S mRNA expression in (C) Huh7 and (F) Hep3B cells with vector or OE‐UBE2S. Asterisks indicate *p*‐value thresholds: ∗∗∗*p* < 0.001.

### 3.8. UBE2S Inhibits Ferroptosis in HCC Cells and Is Regulated by Sorafenib

UBE2S protein levels showed a time‐dependent decline following sorafenib treatment (Figure 9A–B), suggesting a link between UBE2S downregulation and ferroptotic activation. To validate this mechanism, we performed rescue experiments in UBE2S‐overexpressing Huh7 and Hep3B cells treated with sorafenib. Sorafenib alone significantly increased intracellular Fe^2+^ and MDA levels relative to vector controls (Figure 9C–D), along with elevated ROS (Figure 9E) and reduced GSH (Figure 9F). However, UBE2S overexpression reversed these sorafenib‐induced alterations (*p* < 0.001) (Figure 9C–F).

**Figure 9:**
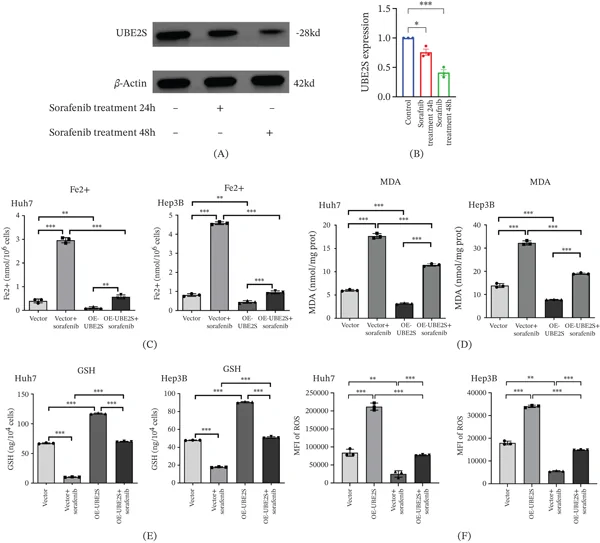
UBE2S inhibits ferroptosis in HCC cells and is regulated by sorafenib. (A) UBE2S protein levels in Huh7 cells at 0, 24, and 48 h after sorafenib treatment. (B) Quantitative analysis of UBE2S protein expression. (C–F) Fe^2+^, MDA, GSH, and ROS levels in Huh7 and Hep3B cells with UBE2S overexpression (OE‐UBE2S) versus vector control, with or without sorafenib treatment. Asterisks indicate *p*‐value thresholds: ∗*p* < 0.05∗∗*p* < 0.01, ∗∗∗*p* < 0.001).

### 3.9. UBE2S Knockdown Combined With Sorafenib Suppresses Tumor Growth in Vivo

To examine UBE2S function in vivo, we generated subcutaneous Hep3B xenografts in nude mice following UBE2S knockdown (LV‐shUBE2S1) (Figure 10A–B). Combined treatment with sorafenib significantly reduced tumor growth compared with LV‐shNC controls (*p* < 0.05) (Figure 10C–F). IHC staining was performed to examine UBE2S and ferroptosis‐associated proteins, namely SLC7A11, GPX4, ACSL4, and TFRC. Compared with the LV‐shNC group, the LV‐shUBE2S1 + sorafenib group exhibited significantly reduced SLC7A11 and GPX4 (antiferroptotic proteins), while concomitantly showing elevated ACSL4 and TFRC (proferroptotic proteins) (Figure 11A–F).

**Figure 10:**
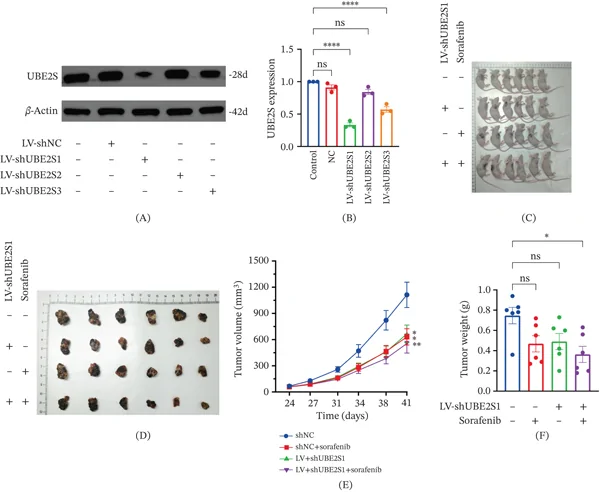
UBE2S knockdown combined with sorafenib suppresses tumor growth in vivo. (A) UBE2S protein levels in Hep3B cells following knockdown. (B) Quantitative analysis of UBE2S protein levels after knockdown. (C–D) Representative photographs of nude mice and excised tumors from each treatment group. (E) Tumor growth curves over time. (F) Final tumor weights at sacrifice. Asterisks indicate *p*‐value thresholds: ns, not significant; ∗*p* < 0.05, ∗∗∗*p* < 0.001.

**Figure 11:**
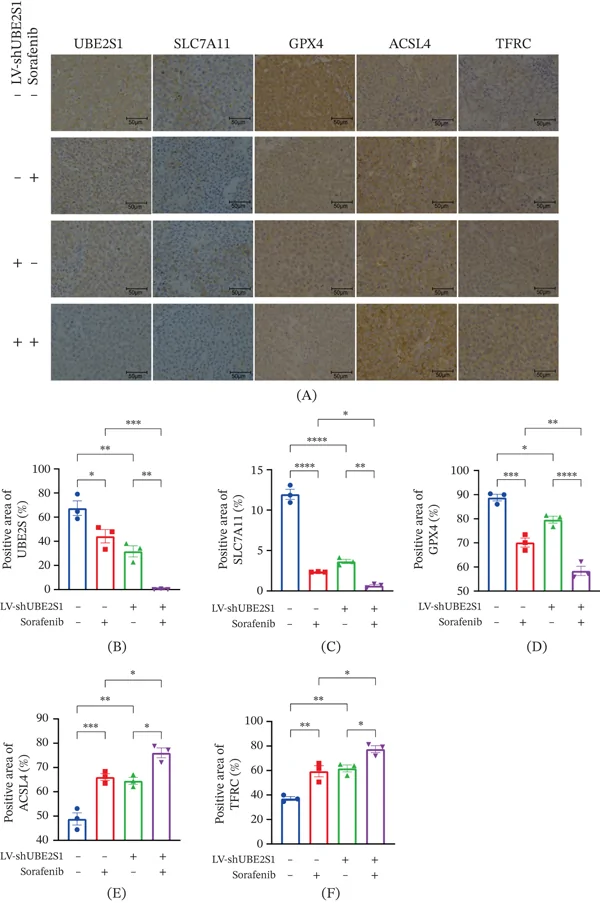
UBE2S knockdown combined with sorafenib modulates ferroptosis‐related protein expression in vivo. (A) Representative IHC staining images of UBE2S, SLC7A11, GPX4, ACSL4, and TFRC in xenograft tumors from the indicated treatment groups. (B–F) Quantitative analysis of IHC staining intensity for (B) UBE2S, (C) SLC7A11, (D) GPX4, (E) ACSL4, and (F) TFRC across treatment groups. Asterisks indicate *p*‐value thresholds: ∗*p* < 0.05, ∗∗*p* < 0.01, ∗∗∗*p* < 0.001.

## 4. Discussion

UBE2S overexpression promotes cell cycle progression through ubiquitin‐mediated p27 degradation, correlating with poor HCC survival [24]. Additionally, UBE2S drives HCC progression via the VHL/HIF‐1*α* and VHL/JAK2/STAT3 signaling pathways [28]. In our study, UBE2S expression did not differ significantly by gender or age, whereas significant differences were observed in T stage, pathological stage, and G stage. Survival curves, ROC curves, and calibration curves further confirmed the prognostic value of UBE2S for 1‐, 2‐, and 3‐year survival in HCC. These findings suggest UBE2S as a prognostic indicator tightly linked to key clinicopathological features in HCC.

Meanwhile, high UBE2S expression, which predicted poor prognosis in our model, was associated with an immunosuppressive tumor microenvironment characterized by increased Tregs and M0 macrophages, along with a decrease in resting memory CD4^+^ T cells. Prior evidence supports these associations, as elevated Tregs independently predict poor HCC prognosis [29], UBE2S expression correlates with macrophage infiltration and adverse outcomes across cancers [30], and reduced resting memory CD4^+^ T cells are linked to adverse outcomes in HCC [31]. It should be emphasized that these associations are derived from bioinformatic predictions and are not causative. Nevertheless, our findings suggest that UBE2S may be linked to adverse outcomes through its association with immune infiltration patterns, although whether UBE2S actively drives immune remodeling requires functional verification. Our analysis further revealed that UBE2S is predominantly enriched in malignant HCC cells, endothelial cells, and plasma cells, which are known to modulate the tumor immune microenvironment through cytokine secretion, antigen presentation, and vascular remodeling [32]. However, any inference regarding such indirect immunomodulatory effects remains speculative without experimental evidence. Collectively, these findings support a predictive association between UBE2S and an immunosuppressive tumor microenvironment, though direct causal contributions remain to be functionally validated.

In our study, the Low‐UBE2S group showed marked upregulation of proferroptotic genes, whereas the High‐UBE2S group displayed preferential elevation of antiferroptotic genes. Consistently, ssGSEA revealed lower enrichment scores for ferroptosis and oxidative stress in the high‐UBE2S group. UBE2S exerts oncogenic effects by promoting p53 ubiquitination [26], and given that p53 plays a key role in ferroptosis, this observation suggests a potential mechanistic connection between UBE2S and ferroptosis‐related prognosis in HCC [27].

Furthermore, we constructed a new HCC prognostic model designated as UFP consisting of CENPA and PAGE1. CENPA inhibits ferroptosis via STMN1 upregulation, thus facilitating HCC growth [33]. PAGE1 may contribute to tumor development in multiple cancer types [34]. PAGE4, which belongs to the same family as PAGE1, protects cells from oxidative damage by increasing p21 and inhibiting ROS production [35]. These findings underscore the potential involvement of CENPA and PAGE1 in ferroptosis regulation and HCC progression. The precise mechanisms underlying these effects warrant further investigation.

Currently, cancer prognosis prediction in clinical practice mainly relies on traditional clinical factors such as gender, age, pathological stage, G stage, and imaging analysis results; however, the clinical outcomes of HCC remain suboptimal, primarily attributed to the substantial interpatient heterogeneity observed in this population [36]. Our UFP model demonstrates superior predictive performance compared with conventional clinical parameters reported in prior studies; however, due to limitations in sample size and type, whether UFP is superior to some commonly used clinical features, such as ultrasound imaging diagnosis, AFP levels, and viral hepatitis [37], requires further analysis and verification. A nomogram quantifies an individual patient′s survival probability based on the cumulative scores of each included clinical variable [38]. The nomogram constructed in this study exhibits high accuracy and can provide clinicians with assistance in personalized diagnosis and treatment of HCC. Moreover, the use of ICGC datasets and single‐cell datasets as external validation sets yielded results consistent with TCGA analysis. However, as a bulk RNA‐seq‐based model, it lacks spatial resolution and cannot capture single‐cell heterogeneity. Future integration of spatial transcriptomics methods, such as scLM for single‐cell consensus clustering [39] and SpaIM for spatial imputation [40], may further enhance predictive accuracy and clinical applicability by overcoming these limitations.

Building upon these bioinformatic findings, we experimentally validated that UBE2S deficiency promotes iron overload and lipid peroxidation, whereas UBE2S overexpression protects HCC cells against erastin, a canonical ferroptosis inducer that inhibits system Xc^−^ to cause GSH exhaustion [41]. These results establish UBE2S as a ferroptosis inhibitor in HCC. Our Western blot data revealed that UBE2S positively modulates the protein levels of GPX4 and SLC7A11, two paramount gatekeepers of the cellular antioxidant system [42]. Notably, Liu et al. reported that the deubiquitylase OTUB1 stabilizes SLC7A11 by removing its ubiquitin chains, thereby suppressing ferroptosis in cancer cells [43]. Based on this, we speculate that UBE2S may indirectly enhance SLC7A11 and GPX4 expression by modulating the activity or stability of OTUB1 or functionally related deubiquitylases. Nevertheless, this putative mechanism requires direct experimental validation in future studies.

Our experimental data revealed that sorafenib treatment downregulated UBE2S expression in HCC cells, and overexpression of UBE2S attenuated sorafenib‐induced accumulation of Fe^2+^, MDA, and ROS, while restoring GSH levels. These observations indicate that sorafenib may exert its ferroptotic effect, at least partially, through downregulation of UBE2S. Notably, MT‐1G has been shown to facilitate sorafenib resistance by suppressing ferroptosis in HCC cells [44]. Given the parallels with UBE2S‐mediated ferroptosis suppression, targeting UBE2S may offer a complementary approach to counteract sorafenib resistance. In xenograft models, UBE2S knockdown combined with sorafenib significantly reduced tumor volume and weight. IHC analysis revealed marked suppression of the antiferroptotic factors SLC7A11 and GPX4, whereas the proferroptotic mediators ACSL4 and TFRC were significantly elevated. Lipid peroxides are converted into nontoxic alcohols by GPX4, thereby suppressing ferroptosis [45]; SLC7A11 promotes GSH synthesis by cystine uptake, indirectly enhancing GPX4 activity and suppressing ferroptosis [46]. Conversely, ACSL4 promotes arachidonoyl‐CoA biosynthesis and phospholipid peroxidation, thereby promoting ferroptosis [47], whereas TFRC facilitates iron uptake and enhances ferroptosis sensitivity [48]. Altogether, our data suggest that UBE2S acts as a ferroptosis suppressor in HCC and may serve as a promising candidate for therapeutic intervention. Nevertheless, we acknowledge that the current in vivo evidence is indirect, as direct ferroptosis markers, including lipid peroxidation and iron accumulation, were not measured in the xenograft models. Thus, the causal link between UBE2S knockdown and ferroptosis‐driven tumor suppression in vivo requires further validation.

In conclusion, the UFP, constructed from UBE2S‐related ferroptosis genes, serves as an independent prognostic factor and demonstrates high accuracy and practical utility in predicting survival outcomes for HCC patients. Both in vitro and in vivo data confirmed that UBE2S suppresses ferroptosis through upregulation of SLC7A11 and GPX4 expression. Furthermore, xenograft tumor growth in nude mice was significantly inhibited by UBE2S knockdown combined with sorafenib, suggesting that UBE2S upregulation may contribute to sorafenib resistance. Future studies will prioritize direct in vivo measurement of ferroptosis markers and further assess the therapeutic efficacy of combined UBE2S knockdown and sorafenib in clinically relevant HCC models, including patient‐derived xenografts and genetically engineered mouse models. Collectively, this study provides a robust prognostic tool for HCC and identifies UBE2S as a promising therapeutic target for this disease.

## Author Contributions

Zhijing Mo, Xiaoyan Wang, and Kangzhi Li designed and directed the research. Hao Zhang and JiaJun Yao contributed to the analysis and interpretation of data and wrote the main manuscript text. Hao Zhang, JiaJun Yao, Jun Cao, Zehao Guo, and Wendong Jiang performed the experiments and analyzed results. Hao Zhang and JiaJun Yao have contributed equally to this work and share first authorship.

## Funding

This study was supported by the National Natural Science Foundation of China, 10.13039/501100001809, No. 32360171, and the Guangxi Natural Science Foundation, No. 2024GXNSFAA010362.

## Disclosure

All authors read and approved the final manuscript.

## Ethics Statement

The use of animal tissues and all experimental procedures were approved by the Animal Ethics Committee of Guilin Medical University (Approval No.: GLMC202003010) and performed in accordance with institutional guidelines and relevant national regulations for animal welfare.

## Consent

The authors have nothing to report.

## Conflicts of Interest

The authors declare no conflicts of interest.

## Data Availability

The datasets used and/or analyzed during the current study are available from the corresponding author on reasonable request.
